# Improvement in persistent idiopathic facial pain with comorbid ADHD using the combination of a dopamine system stabilizer and psychostimulant: A case report

**DOI:** 10.1002/ccr3.7552

**Published:** 2023-06-19

**Authors:** Satoshi Kasahara, Yuichi Kato, Kaori Takahashi, Ko Matsudaira, Naoko Sato, Ken‐Ichi Fukuda, Akira Toyofuku, Shin‐Ichi Niwa, Kanji Uchida

**Affiliations:** ^1^ Department of Anesthesiology and Pain Relief Center The University of Tokyo Hospital Tokyo Japan; ^2^ Department of Pain Medicine Fukushima Medical University School of Medicine Fukushima Japan; ^3^ Department of Pediatric Dentistry, School of Life Dentistry at Tokyo Nippon Dental University Tokyo Japan; ^4^ Department of Dental Anesthesiology Tokyo Dental College Tokyo Japan; ^5^ Nursing Department The University of Tokyo Hospital Tokyo Japan; ^6^ Division of Special Needs Dentistry and Orofacial Pain Tokyo Dental College Tokyo Japan; ^7^ Department of Psychosomatic Dentistry, Graduate School of Medical and Dental Sciences Tokyo Medical and Dental University Tokyo Japan; ^8^ Department of Psychiatry, Aizu Medical Center Fukushima Medical University Aizuwakamatsu Japan

**Keywords:** aripiprazole, attention‐deficit hyperactivity disorder, dopamine, methylphenidate, pain behavior, persistent idiopathic facial pain

## Abstract

**Key Clinical Message:**

Persistent idiopathic facial pain (PIFP) and attention‐deficit/hyperactivity disorder (ADHD) may coexist and can be improved with ADHD medications. Thus, clinicians should screen for ADHD by a multidisciplinary approach when treating PIFP and differentiate between other odontogenic disorders.

**Abstract:**

We report a case of a woman with persistent idiopathic facial pain (PIFP) and attention‐deficit/hyperactivity disorder (ADHD) that markedly improved with the administration of a combination of aripiprazole (APZ) and methylphenidate (MP) treatment. Screening for ADHD and administration of APZ and/or MP may be considered in treating PIFP.

## Graphical abstract

Aripiprazole, a dopamine system stabilizer, and methylphenidate, a dopamine transporter reuptake inhibitor psychostimulant, dramatically improved pain behavior in persistent idiopathic facial pain.

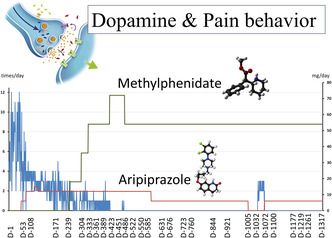

## 1 INTRODUCTION

Persistent idiopathic facial pain (PIFP) is defined as persistent facial pain of variable nature characterized by a dull or tingling sensation persisting for more than 2 h a day for >3 months. Moreover, PIFP has no clear clinical localization and does not correspond with the innervation of peripheral nerves. A neurological dropout syndrome or local cause has been ruled out.^1^ Additionally, PIFP, burning mouth syndrome (BMS), and persistent idiopathic dentoalveolar pain (PIDAP) are considered a subclass of idiopathic orofacial pain.

Persistent idiopathic facial pain is a relatively rare disorder, with a prevalence of 0.03% in the general population.^2^ However, it is relatively common in the clinical setting, with a 10%–21% prevalence among patients who visit oral and facial pain clinics.^3^ As PIFP often develops after minor procedures such as dental treatment, patients are more likely to undergo invasive treatments such as tooth extraction to treat the pain, which often leads to the extraction of many healthy teeth without improvement, resulting in a vicious cycle.^4^

Recently, attention‐deficit/hyperactivity disorder (ADHD), a neurodevelopmental disorder, has been associated with chronic pain.^5^ ADHD is a chronic condition that can cause functional impairment across various domains of daily life for an extended period of time.^6^ Additionally, ADHD can be associated with migraine,^7^ fibromyalgia,^8^, ^9^, ^10^ chronic lower back pain,^11^, ^12^, ^13^ and PIDAP,^14^ which are classified as primary pain syndromes in the 11th revision of the International Statistical Classification of Diseases,^15^ suggesting that ADHD is likely to be a comorbidity of chronic primary pain. As PIFP is classified as a primary pain syndrome and both PIFP and ADHD may involve dopamine system dysfunction,^16^, ^17^ it is possible for these two conditions to coexist.^18^ However, there are no reports on the association between PIFP and ADHD.

We herein report a case with PIFP and ADHD that improved dramatically with the administration of a combination of aripiprazole (APZ) (dopamine system stabilizer) and methylphenidate (MP) (ADHD medication), both of which improve dopamine system neurotransmission.

## 2 CASE PRESENTATION

### 2.1 Patient

The patient in this case study was a 65‐year‐old Japanese woman with a height of 160 cm and a weight of 50 kg. She experienced oral pain and stiffness in the neck and jaw following the removal surgery of a nasal prosthesis in 2008. The patient had been treated without any improvement at seven different medical facilities; treatment included conservative therapies, amoxapine, duloxetine, and benzodiazepine. Due to the pain, the patient had difficulties performing her daily activities, including volunteering and nursing care work, which she did until age 58 when the symptoms forced her to discontinue working.

**FIGURE 1:**
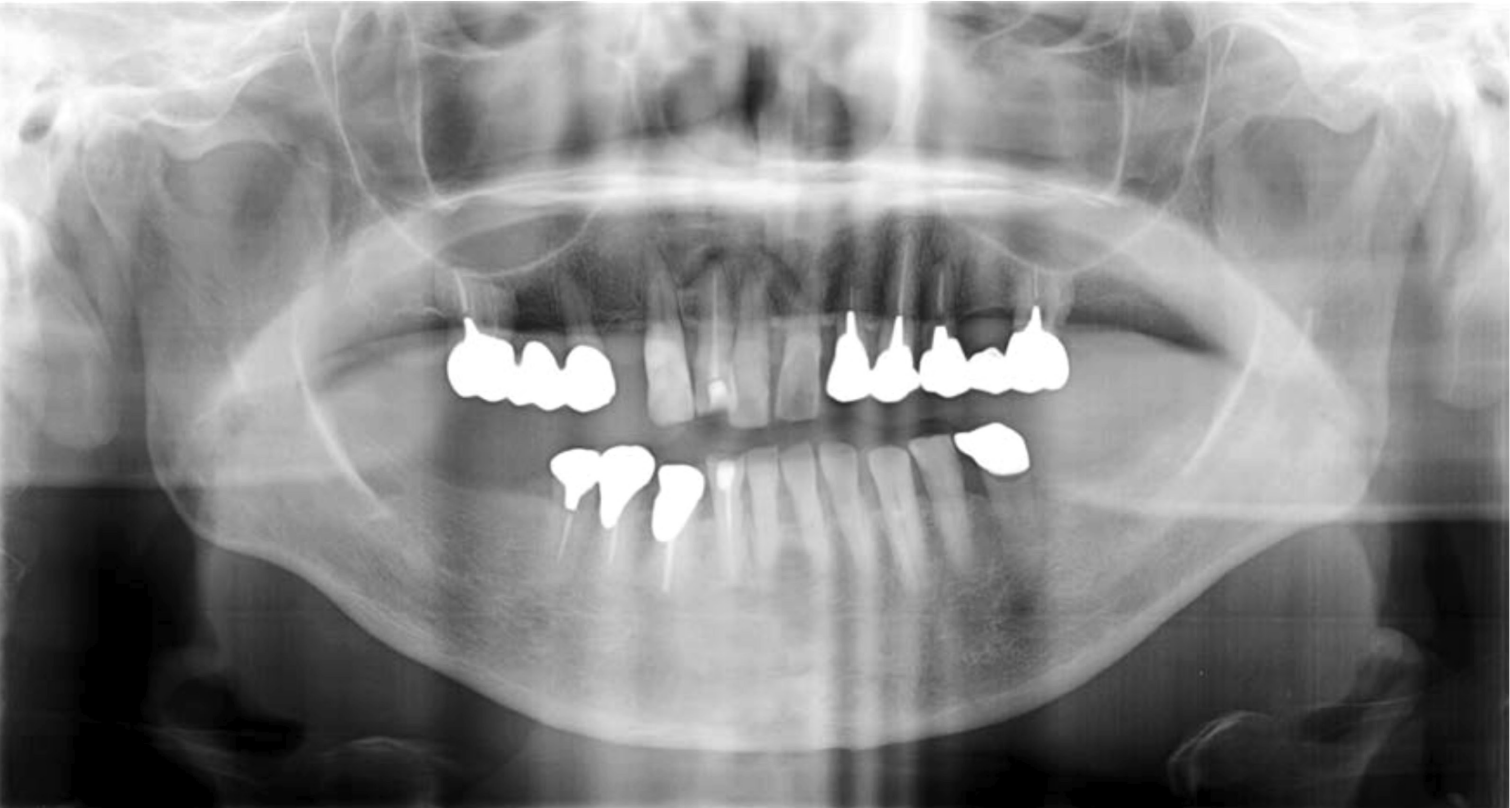
White areas with high x‐ray absorption indicate cast crowns and pontics. No dental abnormalities that could explain the patient's persistent idiopathic facial pain were found.

In February 2010, the patient visited the Department of General Medicine, Nippon Dental University Hospital, a tertiary care institution specializing in oral and facial pain. There were no findings of infection at the site where the nasal prosthesis was removed. Head radiography revealed mild‐to‐moderate alveolar bone resorption in the whole jaw and permeation in the right maxillary central incisor, first molar, and left second maxillary premolar root apex (Figure 1). The patient was diagnosed with moderate periodontitis and apical periodontitis of the right maxillary central incisor, first molar, and left second maxillary premolar. However, no spontaneous or percussion pain was observed for moderate periodontitis or apical periodontitis, and no organic abnormality corresponding to the chief complaint of intraoral pain and stiffness from the neck to the jaw was observed. Moreover, the pain was beyond the trigeminal nerve range and was dull rather than electric in nature. There was no clear trigger point to infer muscle‐related pain, and the patient was able to eat. Thus, local causes of teeth or maxillofacial pain were ruled out, and the pain was considered to be related to the central nervous system.

The patient was diagnosed with PIFP and treated at Nippon Dental University Hospital with amitriptyline, pregabalin, clonazepam, risperidone, and olanzapine for 5 years. However, the pain did not improve with those drugs, and the medication was discontinued; she was referred to a psychiatrist, SK, at the Pain Clinic of Tokyo University Hospital in June 2015 due to a suspected somatoform disorder and the need for a multidisciplinary approach in collaboration with a psychiatrist. The patient lives with her husband, who was always present during examinations. She usually complained of jaw stiffness and pain; whenever she felt pain, she asked her husband to check her jaw repeatedly (more than 10 times daily). The average pain numerical rating scale (NRS)^19^ score at the first visit was 4/10. The Hospital Anxiety and Depression Scale‐Anxiety (HADS‐A)^20^ score was 10/21, and the HADS‐Depression (HADS‐D) score was 9/21. The Mini‐International Neuropsychiatric Interview^21^ was conducted to differentiate comorbid psychiatric disorders. The patient had never had a major depressive episode with hypochondriac delusions or other melancholy‐type features (manic or psychotic). Additionally, obsessive‐compulsive disorder (OCD) was ruled out, as the repeated check requests to her husband were for seeking medical help and reassurance, rather than for ignoring or suppressing any frightening images of her jaw ailment. Accordingly, based on the Diagnostic and Statistical Manual of Mental Disorders‐5 (DSM‐5) diagnostic criteria,^6^ she was diagnosed with somatic symptom disorder. During the examination, the patient frequently fiddled with her hair while speaking, interrupted her husband while he was speaking, and corrected his comments.

### 2.2 Interventions

With her consent, her husband was asked to record the number of times she asked him to check her jaw each day, which was reported back to SK during each visit. This number was used as an indicator of pain behavior, and its trend is shown in Figure 1. The patient visited our pain clinic approximately once a month and answered the pain NRS,^19^ Euro QoL 5 Dimension (EQ5‐D),^22^, ^23^ pain disability assessment scale (PDAS),^24^ HADS‐A/D,^20^ and Pain Catastrophizing Scale (PCS)^25^ questionnaires at each visit. Considering the change in NRS scores for chronic pain and the concept of minimum clinically important difference (MCID), a decrease of −2 points (−33.0%) or more in NRS scores was considered substantial or optimal.^26^ Health‐related aspects of quality of life were assessed using the EQ‐5D, with a score of 0 indicating death and 1.0 indicating perfect health^22^, ^23^ (MCID in chronic pain: 0.08).^27^ Disability in daily functioning due to chronic pain was assessed using the PDAS, wherein a score of 10 or higher is considered a clinical level^24^ (MCID: 6.71).^27^ Symptoms of anxiety and depression were assessed using HADS‐A/D. A HADS score of 8 or higher is considered a clinical level^28^ (MCID: 1.5).^29^ Pain‐related catastrophic thoughts were assessed using the PCS (MCID: 6.48 points).^27^ ADHD symptoms were assessed using the long version of the Conners' Adult ADHD Rating Scale (CAARS) self‐report (CAARS‐S) and observer‐rated (CAARS‐O) questionnaires, which were completed by the patient and her husband, respectively.^30^ A patient is considered to have clinical psychiatric‐level ADHD symptoms if any of the eight CAARS subscale scores exceed 65 points. The CAARS assessment was performed twice, once before methylphenidate (MP) administration (Day 239) and again 3 months after the completion of dosage adjustment (Day 723).

### 2.3 Outcomes

**FIGURE 2:**
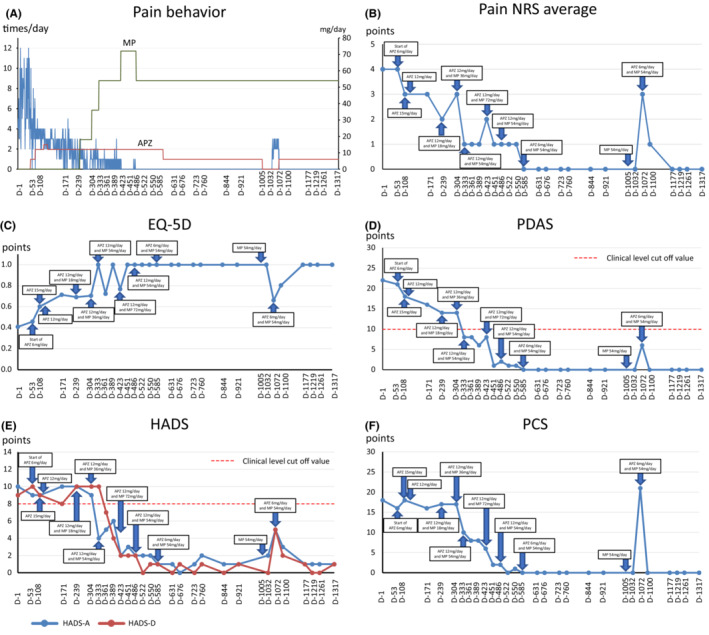
Course of treatment and objective/subjective parameters. (A) Pain behavior; (B) average pain NRS; (C) EQ‐5D; (D) PDAS; (E) HADS; (F) PCS. APZ, aripiprazole; D, day; EQ‐5D, Euro QoL‐5 Dimension; HADS‐A/D, Hospital Anxiety and Depression Scale‐Anxiety/Depression; MP, methylphenidate; NRS, numerical rating scale; PCS, Pain Catastrophizing Scale; PDAS, pain disability assessment scale.

**FIGURE 3:**
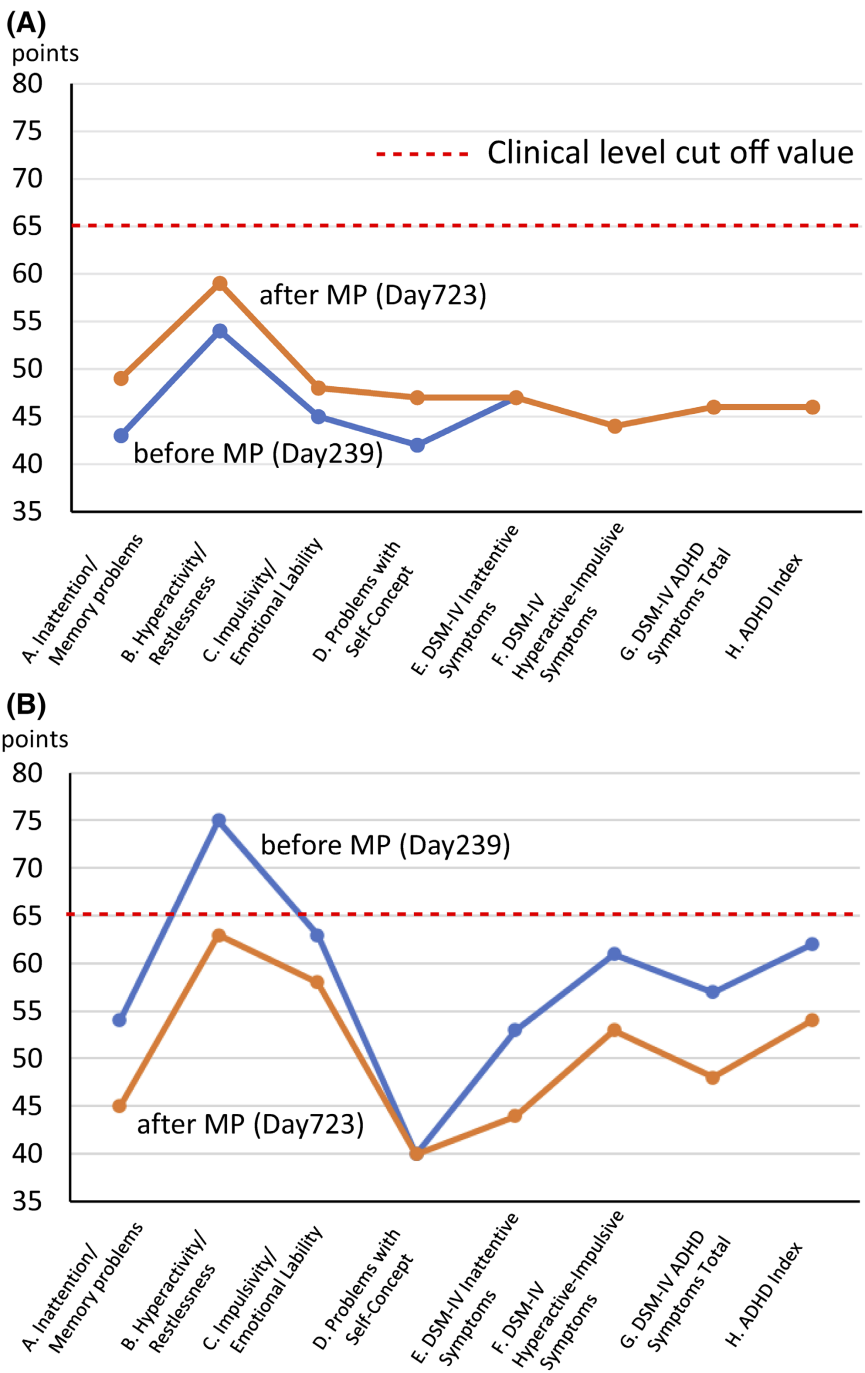
Changes in CAARS scores before and after the administration of methylphenidate. (A) CAARS‐S; (B) CAARS‐O. ADHD, attention‐deficit/hyperactivity disorder; CAARS‐S/O, Conners' Adult ADHD Rating Scale Self‐report/Observer‐rated; DSM‐IV, Diagnostic and Statistical Manual of Mental Disorders, fourth edition.

Trends in pain behavior and NRS, EQ‐5D, PDAS, HADS, and PCS scores are shown in Figure 2. Changes in CAARS‐S/O scores before and after MP administration are shown in Figure 3.

### 2.4 Treatment course and changes in pain behavior

Mean pain behavior prior to pharmacotherapy initiation was 6.8 ± 2.8 times/day. At Day 53, as standard medications for PIFP (tricyclic antidepressants and antiepileptic drugs) had been ineffective in the past, APZ, a dopamine system stabilizer reported to be effective for BMS and PIDAP,^31^, ^32^ was administered (starting dose, 6 mg/day). Subsequently, mean pain behaviors immediately after the first treatment decreased to 4.6 ± 2.3 times/day. Moreover, the patient's motivation improved, and she resumed volunteer activities previously discontinued due to pain. At Day 73, when the APZ dose was increased to 12 mg/day, mean pain behavior decreased to 3.4 ± 0.9 times/day. Furthermore, the patient became more motivated to perform other activities like sewing. At Day 108, when the APZ dose was increased to 15 mg/day, mean pain behavior decreased to 2.8 ± 0.7 times/day. However, drooling, an APZ‐induced extrapyramidal symptom, was observed. At Day 124, APZ was reduced to 12 mg/day, and drooling stopped. Thereafter, APZ was continued at 12 mg/day; however, pain behavior persisted (2.1 ± 1.1 times/day). This did not lead to remission, and the improvement in pain behavior with APZ therapy plateaued.

On Day 171, SK became aware of the possibility of ADHD comorbidity based on the patient's frequent fiddling with her own hair and interrupting her husband's comments during the examination. The patient was then interviewed in detail about her developmental history and shared episodes suggestive of ADHD, such as her habit of climbing trees since childhood, her preference for climbing to high places even as an adult, frequent falls and constant injuries, and frequent forgetfulness during elementary school. Moreover, the patient's husband reported that the patient is usually calm but likes to drive fast and tends to lose her temper when another car cuts her off, which has often been observed since her youth and seems to be a complete personality change. As these findings were considered suggestive of ADHD, CAARS‐S/O was administered (Figure 2). The patient's own CAARS‐S scores were in the normal range; however, her husband's CAARS‐O score was B with hyperactivity/restlessness at 75, indicating clinical‐level ADHD hyperactivity symptoms. Based on interviews with her and her husband, the patient met all nine DSM‐5 ADHD inattention items (“making careless mistakes,” “difficulty sustaining attention in tasks,” “poor listening to others,” “often does not follow through on instructions,” “difficulty organizing tasks,” “avoiding tasks requiring sustained mental effort,” “lose things necessary for tasks,” “easily distracted by ambient noise,” and “forgetful in daily activities”). The patient also met 6/9 of the ADHD hyperactivity/impulsivity items (“fidgets with hands,” “feeling restless,” “unable to engage in leisure activities quietly,” “acting as if driven by a motor”, “cannot wait for turn in the conversation,” and “often interrupt others”). Therefore, she was also diagnosed with a combined type of ADHD.

On Day 239, MP was started at 18 mg/day, and mean pain behavior decreased to 0.9 ± 0.9 times/day. On Day 304, MP was increased to 36 mg/day, and pain behavior decreased to 1.1 ± 0.7 times/day. On Day 333, MP was increased to 54 mg/day, and pain behavior was reduced to 0.6 ± 0.6 times/day. The patient also stated that she had started taking drawing and flower arrangement classes and was enjoying her daily life. On Day 423, MP was administered at 72 mg/day, and pain behavior decreased to 0.1 ± 0.3 times/day. The patient reported not feeling much change after 72 mg/day of MP; hence, the dose was reduced at Day 486 to 54 mg/day. On Day 585, APZ was administered at 6 mg/day; however, the pain behavior remained in remission at 0.0 ± 0/0 times/day. As the patient's condition was stable afterward, APZ was discontinued on Day 1005. Approximately 1 month later, the pain behavior increased to 0.9 ± 1.2 times/day. Therefore, at Day 1072, APZ was restarted at 6 mg/day, and the pain behavior disappeared completely (0.0 ± 0.0 times/day). Subsequently, the patient was in remission again. As a result, the patient has reentered the nursing profession and is back to her original healthy state (before pain development), according to her husband.

### 2.5 Pain‐related scale changes

Gradual improvement in pain NRS, PDAS, and EQ‐5D, but not HADS‐A/D and PCS scores, was observed in response to APZ. However, when MP was used in combination on Day 239, dramatic improvement in EQ‐5D, HADS‐A/D, and PCS scores were observed. When APZ (6 mg/day) was discontinued at Day 1005, all pain‐related scale scores temporarily worsened; however, when APZ (6 mg/day) was resumed on Day 1072, all pain‐related scale scores improved, and the patient was again in remission.

The respective changes from baseline to Day 723 were 4 points for pain NRS, 0.6 points for EQ‐5D, 22 points for PDAS, 9 points for HADS‐A, 8 points for HADS‐D, and 18 points for PCS, showing improvements over MCID for all scales.

### 2.6 ADHD scale changes

The patient's CAARS‐S scores were in the normal range at baseline before (Day 239) and after (Day 723) MP administration. At baseline (Day 239), the score of subscale B (the hyperactivity/restlessness) was 75 based on the husband's CAARS‐O response; however, after MP administration (Day 723), the overall profile improved, and the subscale B (hyperactivity/restlessness) score improved to 63 points, which was below the clinical level of 65 points.

## 3 DISCUSSION

This case demonstrated the following points: (1) PIFP can be comorbid with ADHD; and (2) PIFP can be in remission when APZ and MP are used in combination.

To the best of our knowledge, there are no reports on the coexistence of PIFP and ADHD, making this the first report of such a case. It is not easy to recognize ADHD characteristics at first glance as adults with ADHD often compensate and adapt to its symptoms; hence, even in psychiatric practice, reportedly, more than 80% of cases of adult ADHD are overlooked during diagnosis.^33^ As patients with PIFP are usually treated by dentists unfamiliar with ADHD, it is assumed that ADHD as a comorbidity with PIFP has not previously been sufficiently recognized or focused on as pain‐related comorbidity. In this case, the psychiatrist had initially overlooked the coexistence of ADHD. During treatment, the possibility of ADHD coexistence was recognized, and a diagnosis of ADHD was made after a close examination that included information on the patient's ADHD symptoms obtained from family members.

Moreover, to the best of our knowledge, there have been no reports on the efficacy of the combination of APZ and MP in treating chronic pain (PIFP) with ADHD, and this is the first report of such a case. Regarding drug therapy for PIFP, antidepressants, and antiepileptic drugs have been reported to have beneficial effects.^3^, ^4^ However, most of these recommendations are based only on expert opinion; hence, treatments must be developed based on a better understanding of the pathophysiology of PIFP.^3^, ^4^ In the present case, as has been previously reported for BMS and PIDAP,^31^, ^32^ APZ produced a significant improvement in PIFP; however, when the dose was increased to 15 mg/day, the side effect of drooling, an extrapyramidal symptom, appeared. Thus, the dose had to be reduced to 12 mg/day, and PIFP improvement plateaued. Subsequently, when APZ (6 mg/day) was stopped to transition to MP monotherapy, PIFP flared up; hence, APZ (6 mg/day) was restarted, and PIFP disappeared. This treatment course suggests that both APZ and MP were necessary to bring PIFP to remission in this case.

Aripiprazole acts as a partial agonist for dopamine D2 and D3 receptors and is called a dopamine system stabilizer.^34^ APZ also acts as a partial serotonin (5‐HT)1A receptor agonist and 5‐HT2A inhibitor, enhancing dopamine neurotransmission.^34^ MP also acts on presynaptic dopamine (DA‐T) and noradrenergic (NA‐T) transporters to inhibit reuptake and increase the concentration of dopamine and norepinephrine in the synaptic cleft.^34^ The affinity of MP for DA‐T is 10‐fold higher than for NA‐T, and it is more selective for dopamine.^35^ Therefore, theoretically, both MP and APZ commonly enhanced dopaminergic neurotransmission during treatment, suggesting dopamine system dysfunction as a pathophysiology of PIFP. This clinically replicated the results of a previous PET study.^17^ Thus, two pharmacological interventions, a dopamine system stabilizer, and a DA‐T reuptake inhibitor psychostimulant, may be options as treatment strategies for dopamine‐related nervous system dysfunction in PIFP. In PIFP with comorbid ADHD, monotherapy with APZ, MP, or a combination of the two, may be considered.

As this case demonstrated, PIFP can be comorbid with ADHD and can be treated with a combination of APZ and MP, drugs that enhance dopamine neurotransmission. Therefore, screening for ADHD and administering APZ and MP may be considered in PIFP cases. However, there are three limitations to this study. First, physicians are often reluctant to prescribe stimulants for chronic pain because of the risk of dependence, abuse, hypertension, and tachycardia.^36^ Therefore, MP should only be prescribed to patients with a confirmed diagnosis of ADHD, and physicians should monitor for abnormal stimulant‐related behavior through regular office visits and check for residual medication and usage and for cardiovascular side effects. Second, it could be possible that the patient in this case also had OCD, although we did rule out OCD based on the DSM‐5. APZ has been reported to be effective as a single agent for OCD,^37^ and the possibility that APZ may have ameliorated compulsive behavior expressed as pain behavior should be considered. Third, MP also has an inhibitory effect on NA‐T, albeit at approximately 1/10th of the effect on DA‐T^35^; thus, the improvement of NA neurotransmission may have contributed to the therapeutic effect in this case.

In the future, it would be advisable to examine ADHD as a comorbidity in PIFP and verify any associations and causal relationships between pain symptoms and ADHD in studies with larger sample sizes.

## AUTHOR CONTRIBUTIONS

**Satoshi Kasahara:** Conceptualization; data curation; funding acquisition; project administration; writing – original draft. **Yuichi Kato:** Data curation. **Kaori Takahashi:** Funding acquisition. **Ko Matsudaira:** Conceptualization; funding acquisition. **Naoko Sato:** Data curation. **Ken‐Ichi Fukuda:** Conceptualization; funding acquisition. **Akira Toyofuku:** Conceptualization; funding acquisition. **Shin‐Ichi Niwa:** Supervision. **Kanji Uchida:** Supervision.

## FUNDING INFORMATION

This study was supported by a Grant‐in‐Aid for Scientific Research (C), No. 20K07755 from the Japan Society for the Promotion of Science. The funder had no role in the design of the study or collection, analysis, or interpretation of data or in writing the manuscript.

## CONFLICT OF INTEREST STATEMENT

The authors declare no conflicts of interest.

## ETHICS STATEMENT

This case report was approved by the Research Ethics Committee of Tokyo University Hospital (approval no. 3678).

## CONSENT

Verbal informed consent for participation in this study was obtained from the patient. Written informed consent was obtained from the patient for publication of this case report and any accompanying image.

## Data Availability

Data sharing are not applicable to this article as no new data were created or analyzed in this study.
